# Unexpected Behavior of Some Nitric Oxide Modulators under Cadmium Excess in Plant Tissue

**DOI:** 10.1371/journal.pone.0091685

**Published:** 2014-03-13

**Authors:** Jozef Kováčik, Petr Babula, Bořivoj Klejdus, Josef Hedbavny, Markéta Jarošová

**Affiliations:** 1 Institute of Chemistry and Biochemistry, Faculty of Agronomy, Mendel University in Brno, Brno, Czech Republic; 2 CEITEC – Central European Institute of Technology, Mendel University in Brno, Brno, Czech Republic; 3 Department of Natural Drugs, Faculty of Pharmacy, University of Veterinary and Pharmaceutical Sciences Brno, Brno, Czech Republic; Universidade Federal de Vicosa, Brazil

## Abstract

Various nitric oxide modulators (NO donors - SNP, GSNO, DEA NONOate and scavengers – PTIO, cPTIO) were tested to highlight the role of NO under Cd excess in various ontogenetic stages of chamomile (*Matricaria chamomilla*). Surprisingly, compared to Cd alone, SNP and PTIO elevated Cd uptake (confirmed also by PhenGreen staining) but depleted glutathione (partially ascorbic acid) and phytochelatins PC_2_ and PC_3_ in both older plants (cultured hydroponically) and seedlings (cultured in deionised water). Despite these anomalous impacts, fluorescence staining of NO and ROS confirmed predictable assumptions and revealed reciprocal changes (decrease in NO but increase in ROS after PTIO addition and the opposite after SNP application). Subsequent tests using alternative modulators and seedlings confirmed changes to NO and ROS after application of GSNO and DEA NONOate as mentioned above for SNP while cPTIO altered only NO level (depletion). On the contrary to SNP and PTIO, GSNO, DEA NONOate and cPTIO did not elevate Cd content and phytochelatins (PC_2_, PC_3_) were rather elevated. These data provide evidence that various NO modulators are useful in terms of NO and ROS manipulation but interactions with intact plants affect metal uptake and must therefore be used with caution. In this view, cPTIO and DEA NONOate revealed the less pronounced side impacts and are recommended as suitable NO scavenger/donor in plant physiological studies under Cd excess.

## Introduction

Nitric oxide (NO) is small gaseous molecule with far-reaching action in living organisms including plants [1]. It affects basic physiology including growth and hormonal balance as well as tolerance to various stress impacts including excess of metals [2]. Metallic stress usually alters NO content in both vascular [3] and non-vascular plants [4] and can contribute to amelioration of metal-induced negative effects [5], [6].

In addition to alteration of NO level, excess of metals also stimulates increase in reactive oxygen species (ROS) formation, depending on the applied concentration, exposure time and given species [7], [8]. This oxidative stress is controlled by various enzymatic and non-enzymatic antioxidants to prevent cellular damage. Glutathione and ascorbic acid are main antioxidants [9] while phytochelatins (PCs) may effectively chelate free metal ions [10].

Cadmium (Cd) has no known biological function in organisms and usually shows more toxic impacts in comparison with other metals when applied in equal doses [11]. Chamomile (*Matricaria chamomilla* L.) is a widely-used medicinal plant that considerably accumulates mainly Cd in the above-ground biomass at high exogenous concentrations without visible damage [12] and is therefore suitable object for the present investigation.

Studies focused on the role of NO usually use its exogenous modulators among which donor sodium nitroprusside (SNP) and scavenger 2-phenyl-4,4,5,5-tetramethylimidazoline-1-oxyl-3-oxide (PTIO) or its carboxy-derivative (cPTIO) are the most widely used [13]–[16]. Other donors of NO such as S-nitrosoglutathione (GSNO) or “NONOate” derivatives are not widely used in plant studies [17], [18]. Though protective effect of NO under Cd or metallic stress was well documented in various species and using common NO modulators [16], [19]–[21], eventual side impacts of these compounds are only poorly known [4]. Besides, responses of antioxidants and chelators such as glutathione, ascorbic acid and phytochelatins to these modulators are not known in detail. For this purpose, we tested the impact of various NO modulators (NO scavengers PTIO and cPTIO or NO donors SNP, GSNO and diethylamine NONOate) on Cd uptake in chamomile plants cultured in hydroponics or seedlings cultured in deionised water only allowing to compare various culture conditions and ontogenetic stages. Assays of above-mentioned metabolites and extensive fluorescence/confocal microscopy were done to identify eventual side impacts under Cd excess and to verify usefulness of NO modulators in laboratory conditions after prolonged exposure (48 h), considering that life time of some compounds is short while studies of metal uptake usually require days.

## Materials and Methods

### 2.1. Plant Culture, Experimental Design and Statistics

Twenty-one days old seedlings of *Matricaria chamomilla* L. (tetraploid ‘Lutea’, Asteraceae) germinated in sand were placed to Hoagland solution and cultured under laboratory conditions as reported earlier [6]–[8]. In these conditions, plants form basal leaf rosettes only. Plants, that had been cultivated hydroponically during 4 weeks, were used in the experiment and further cultured for 48 h in mentioned Hoagland solution enriched with 60 µM Cd^2+^ (added as CdCl_2_·2½H_2_O, Lachema Brno, Czech Republic) alone or in combination with 60 µM PTIO (2-phenyl-4,4,5,5-tetramethylimidazoline-1-oxyl-3-oxide) or 300 µM SNP (sodium nitroprusside). Control was further cultured in Hoagland solution only and pH was checked to be 6.0 in all variants.

Subsequent experiment aimed to verify observations from hydroponically-cultured plants in chamomile seedlings. Seeds (100) were sown on filter paper placed on glass balls (2 mm in diameter) within Petri dishes with deionised water [22]. Seeds germinated within 2 days and after 5 days of further culture (to obtain enough biomass owing to extremely low biomass per one seedling, ca. 1 mg FW), concentrations of Cd^2+^, PTIO and SNP as mentioned above were applied (prepared in deionised water only). Seedlings were harvested after 48 h of exposure to these treatments and selected parameters were assayed in shoot or root or in whole seedling. Another subsequent experiment with seedlings was focused on the impact of alternative NO modulators (S-nitrosoglutathione/GSNO - 300 µM, diethylamine NONOate/NONO - 300 µM and 2-(4-carboxyphenyl)-4,4,5,5-tetramethylimidazoline-1-oxyl-3-oxide/cPTIO - 60 µM, all from Sigma-Aldrich) under 60 µM Cd^2+^ and identical conditions (culture in deionised water and 48 h of exposure to treatments) on Petri dishes.

For fresh mass-requiring parameters, individual plants were powdered using liquid N_2_ and fresh material was extracted as described below. Dry samples (dried at 75°C to constant weight) were analyzed for mineral nutrients including Cd. Two independent repetitions of the whole experiment were performed in order to check reproducibility. Data were evaluated using ANOVA followed by a Tukey’s test (MINITAB Release 11, Minitab Inc.; State College, Pennsylvania) at *P*<0.05. Number of replications (*n*) in tables/figures denotes individual plants measured for each parameter. For seedlings, biomass within one Petri dish was pooled prior to determination of Cd content or phytochelatins (then *n* = 3 means three individual dishes).

### 2.2. Staining and Quantification of Cd and Minerals

Samples were prepared by mineralization of dry material in the mixture of concentrated ultra-pure HNO_3_ and water using microwave decomposition (Ethos Sel Microwave Extraction Labstation, Milestone Inc.) at 200°C over 1 h. Resulting clear solution was quantitatively placed to glass flasks and diluted to a final volume of 10 ml. Measurements were carried out using an atomic absorption spectrometer AA30 (Varian Ltd., Mulgrave, Australia) and the air-acetylene flame. Blank (mixture of HNO_3_ and water) was also checked to ensure correctness of metal quantifications. Accuracy of metal determinations was verified by the addition of known metal concentrations and accuracy of mineralization and measurement was checked using reference plant material: the detection limit of Cd was 0.5 µg L^−1^ at λ_max_ 228.8 nm and other nutrients were quantified as reported earlier [8], [23].

Cd was stained using fluorescence metal indicator Phen Green SK, Diacetate (Life Technologies, USA) according to manufacturer’s instructions [3]. Primary roots were excised ca. 3 cm below the surface of cultivation solutions in the zone of lateral roots formation. In the shoots, adult leaf’s petioles of similar age from 3 individual plants were stained. Sections were observed using fluorescence microscope Axioscop 40 (Carl Zeiss, Germany) equipped with appropriate set of excitation/emission filters.

### 2.3. Fluorescence and Confocal Microscopy of ROS and NO

For fluorescence microscopy, ROS were stained using CellROX Deep Red Reagent (Life Technologies, USA) and NO/RNS with 2,3-diaminonaphthalene (DAN, Sigma-Aldrich) as reported previously [3], [22]. For comparison, NO was also stained using DAF-DA (4,5-diaminofluorescein diacetate, Life Technologies, USA) that is a cell-permeable highly sensitive indicator of nitric oxide and RNS. Freshly prepared hand-made sections were washed twice in PBS buffer (50 mM, pH 7.2) and incubated in DAF-DA (50 µM) in PBS buffer (50 mM, pH 7.2) for 60 min at room temperature and darkness. After incubation, sections were always washed three times by PBS buffer and observed using fluorescence microscope Axioscop 40 (Carl Zeiss, Germany) equipped with appropriate set of excitation/emission filters.

Confocal microscopy was done on various parts of seedlings to compare tissue localization of signal as well as staining reagents. Namely, above-mentioned DAF-DA and its improved derivative 4-amino-5-methylamino-2′,7′-difluorofluorescein diacetate (DAF-FM DA, Sigma-Aldrich) were compared for NO presence. Working solution of DAF-FM DA was prepared as DAF-DA above. Lipid peroxidation was assayed by BODIPY 581/591 C11 lipid peroxidation sensor (Life Technologies, USA) as reported earlier [22]. Amplex UltraRed (568_Ex_/681_Em_ nm, Life Technologies, USA) is reagent for H_2_O_2_ detection according to manufacturer’s instructions. Samples were incubated in a working solution (50 µl of 10 mM Amplex stock solution, 100 µl of horseradish peroxidase –10 U/ml in 0.05 M PBS buffer, pH 6.0, Sigma-Aldrich, USA and 4.85 ml of reaction buffer –50 mM PBS, pH 6.0) for 30 min at room temperature and darkness and observed using confocal microscope Leica TCS SP8 X (Leica, Germany).

### 2.4. Anatomical Responses to Cd and Modulators

Calcofluor White Stain (CWS), a non-specific fluorochrome that binds with cellulose in the cell walls, was purchased from Sigma Aldrich, USA. Calcofluor White Stain is often used to study regeneration of the cell wall in plant protoplasts, but also for localisation of cellulose in the cell walls [24]. Transversal sections of roots or petioles were incubated in CWS for 45 min in darkness. After incubation, sections were three times washed with distilled water and observed using appropriate set of excitation and emission filters (Axioscop 40, Carl Zeiss, Germany). 0.1% (*w/v*) aqueous solution of acridine orange (Sigma Aldrich, USA) was used to evaluate general anatomy of roots/petioles with respect to compounds that pose autofluorescence – lignin, cutin, and suberin [25]. Sections were incubated for 10 min in darkness at room temperature. After incubation, they were washed three times (5 min) in distilled water and observed using appropriate set of excitation and emission filters (Axioscop 40, Carl Zeiss, Germany).

### 2.5. Assay of Antioxidative Enzymes, Antioxidants and Phytochelatins

Fresh tissue was homogenized with small amount of inert sand using cold mortar and pestle in 50 mM potassium phosphate buffer (pH 7.0, 1 g FW/5 ml). After centrifugation, supernatants were used to measure enzyme activities. Ascorbate peroxidase (APX) and guaiacol peroxidase (GPX) activities were measured as the oxidation of ascorbate and guaiacol at 290 and 470 nm, respectively; glutathione reductase (GR) activity was assayed as the reduction of GSSG at 412 nm as described previously [7], [8].

Reduced (GSH) and oxidized glutathione (GSSG) and ascorbic acid (AsA) were extracted with 0.1 M HCl (0.2 g FW/2 ml) and quantified using LC-MS/MS (Agilent 1200 Series Rapid Resolution LC system coupled on-line to a detector Agilent 6460 Triple quadrupole with Agilent Jet Stream Technologies at m/z values 308/76, 613/231 [26] and 177/95 in positive MRM mode, respectively. Separation was done using column Zorbax SB-C18 50×2.1 mm, 1.8 µm particle size and mobile phase consisting of 0.2% acetic acid and methanol (95∶5). The flow-rate was 0.6 ml/min and column temperature was set at 25°C. Freshly prepared standards were used for calibration and quantification [3].

Phytochelatins were quantified in the same 0.1 M HCl supernatants as mentioned above and it showed higher extraction efficiency than water. Quantification was done by LC-MS/MS Agilent system mentioned above using column Zorbax SB-C18 (50×2.1 mm, particles size 1.8 µm, flow 0.5 ml/min, temperature 35°C) and gradient consisting of 0.2% acetic acid (A) and acetonitrile (B) as A:B –0 min (95∶5), 0.4 min (70∶30), 1 min (50∶50), 1.5–2 min (40∶60), 3 min (95∶5). PC_2_ was detected in ESI positive mode at m/z 540/76 (fragmentor 140 V, collision energy 52 eV) and PC_3_ at m/z 772/76 (fragmentor 156 V, collision energy 76 eV), gas/sheath gas temperature 350°C, gas flow 12 l/min, capillary 4000 V and nozzle voltage 500 V similarly to earlier study [27] and by commercially available standard compounds [28].

## Results and Discussion

### 3.1. Responses of Hydroponically-cultured Plants to Treatments

#### 3.1.1. Both NO modulators elevate Cd uptake

**Figure 1:**
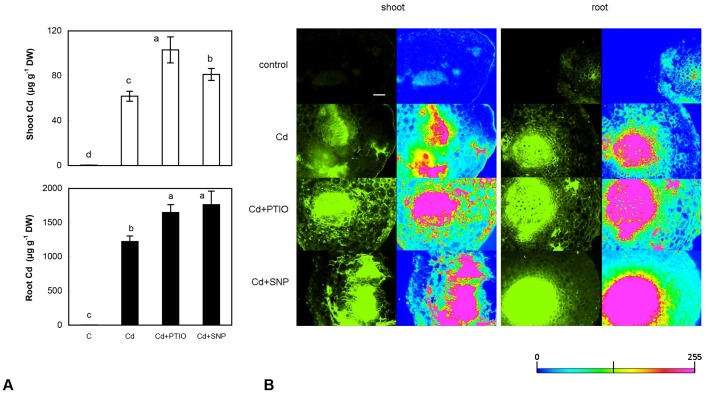
Accumulation of cadmium in chamomile plants (A) and visualization of Cd on freshly-prepared hand-made cross sections from petioles (shoot) and primary roots (root) using Phen Green SK diacetate (B) after 48 h of exposure to Cd alone (60 µM) or with the addition of 2-phenyl-4,4,5,5-tetramethyl-imidazoline-1-oxyl-3-oxide (PTIO, 60 µM) and sodium nitroprusside (SNP, 300 µM). Data are means ± SDs (*n* = 4) for Cd content and three sections were stained for Cd (representative is shown). Values within individual graphs followed by the same letter(s) are not significantly different according to Tukey’s test (*P*<0.05). Shoot means whole above-ground biomass for Cd content; bar indicates 200 µm. Scale bar for concentration map (right column in each panel) indicates intensity of fluorescence signal.

The use of NO modulators in various processes such as metal-induced proline accumulation was studied in detail but quantification of metals is rare [1]. This is the key factor that may affect final response owing to simple assumption that all exogenously applied compounds could exhibit side effects. This is yet more probable during co-application of various modulators under metal excess due to possible chelation: SNP is one such compound because of numerous bonds in the molecule. In accordance, we observed an increase in Cd accumulation in the Cd+SNP treatment in both shoot and root of chamomile (Fig. 1A). This ability of SNP is certainly concentration-dependent and elevated Cd accumulation after higher SNP application (100–500 µM) was also observed in Cd-exposed alga *Scenedesmus* under totally different exposure conditions [4]. It may also be concluded that SNP-enhanced Cd uptake is metal-specific, because other metals revealed rather depletion, e.g. Cu [4] or Ni [14]. Our data agree with those found in tobacco BY-2 cells where even 0.5 µM of SNP increased Cd uptake [16]. Other data contradict this conclusion because *Medicago* roots pre-treated with SNP contained less Cd that could be evoked i) just by pre-treatment instead of co-application and ii) short (6 h) exposure time [15]. The impact of PTIO on metal uptake is less known. PTIO's derivative cPTIO evoked Cd efflux in tobacco BY-2 cells even after 6 h of exposure [16] while our present data showed an increase after PTIO addition (Fig. 1A). This quantitative increase in both SNP and PTIO treatments was qualitatively visualized using metal fluorescence indicator Phen Green though given cross sections represent only a part of total biomass (Fig. 1B). Signal was visible mainly within vascular tissue that is main site for root-to-shoot metal translocation. Fluorescence metal indicators are only rarely used in plant studies. Excluding our previous study [3], we found no data related to staining of cross sections using Phen Green. According to manufacturer, this reagent also reacts with Cu^2+^, Cu^+^, Fe^2+^, Hg^2+^, Pb^2+^, Cd^2+^, Zn^2+^, and Ni^2+^ that could be reason for weak signal in control petioles and roots (Fig. 1B). Analyses of metallic nutrients (Table 1) confirm that Cd-evoked elevation of Phen Green signal is not related to their increase because almost all minerals decreased or were not affected in combined treatments. Only Na content increased in shoots indicating partial uptake from SNP molecule (Table 1). Cd-induced alteration of minerals, despite shorter exposure time, is in accordance with earlier observations in chamomile [8], [12]. Interestingly, both SNP and PTIO ameliorated Cd-induced Zn depletion in roots (Table 1). Methodology of Phen Green use should also be briefly mentioned: upon binding to metal(s), it should loss fluorescence (thus yielding inverse correlation between metal content and fluorescence signal; [29]) but we did not observe such response (Fig. 1). We strongly feel that responses of isolated organelles [29] differ from those of whole tissue preparations where is more difficult to control the level of free metal ions across various types of tissues.

**Table 1:** Quantitative changes of selected mineral nutrients in *Matricaria chamomilla* plants after 48 h of exposure to Cd alone (60 µM) or with the addition of 2-phenyl-4,4,5,5-tetramethylimidazoline-1-oxyl-3-oxide (PTIO, 60 µM) and sodium nitroprusside (SNP, 300 µM).

*Shoot*	control	Cd	Cd+PTIO	Cd+SNP
K (mg g^−1^ DW)	101.5±4.95 a	98.0±2.23 a	94.3±5.17 a	98.9±2.64 a
Na (mg g^−1^ DW)	5.49±0.13 b	5.68±0.36 ab	5.81±0.17 ab	6.25±0.16 a
Ca (mg g^−1^ DW)	5.70±0.27 a	5.74±0.12 a	5.28±0.20 a	5.27±0.13 a
Mg (mg g^−1^ DW)	4.21±0.14 a	4.45±0.20 a	4.08±0.19 a	4.33±0.17 a
Fe (mg g^−1^ DW)	0.17±0.021 a	0.15±0.016 a	0.14±0.017 a	0.16±0.012 a
Zn (µg g^−1^ DW)	48.4±4.17 a	45.9±3.94 ab	38.5±2.38 b	38.2±2.85 b
*Root*				
K (mg g^−1^ DW)	100.2±4.67 a	89.4±2.47 b	77.2±4.10 c	79.1±3.68 c
Na (mg g^−1^ DW)	5.91±0.22 a	5.82±0.27 a	5.96±0.33 a	5.79±0.15 a
Ca (mg g^−1^ DW)	7.58±0.37 a	7.51±0.22 a	7.72±0.29 a	6.60±0.28 b
Mg (mg g^−1^ DW)	2.27±0.14 a	2.10±0.09 ab	2.03±0.15 ab	1.89±0.11 b
Fe (mg g^−1^ DW)	7.60±0.31 a	7.32±0.27 a	6.37±0.24 b	6.09±0.49 b
Zn (µg g^−1^ DW)	78.6±3.06 a	46.7±3.55 c	61.8±2.47 b	67.8±3.72 b

Data are means ± SDs (*n* = 4). Values within rows followed by the same letter(s) are not significantly different according to Tukey’s test (*P*<0.05). Shoot means whole above-ground biomass.

In conclusion, both NO modulators affected Cd uptake being in accordance with other studies. Considering numerous secondary metabolites produced by plants, unlike animals, this could be a reason for species-specific differences mentioned above. However, NO modulator-induced changes should not simply be presented as “NO modulated Cd influx” [16] owing to subsequent impacts observed at the level of antioxidants (see below) that are far from expected “NO modulated” effects.

#### 3.1.2. ROS and NO accumulations revealed reciprocal changes

**Figure 2:**
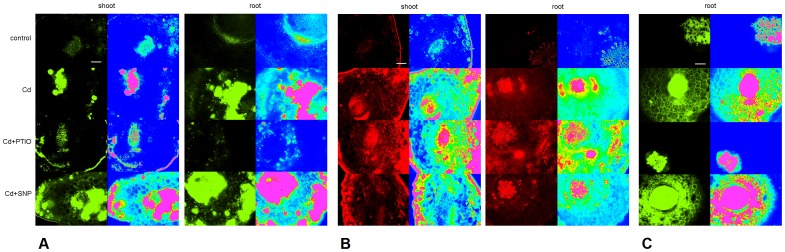
Fluorescence visualization of RNS/NO (stained with 4,5-diaminofluorescein diacetate, A) and ROS (stained with CellROX Deep Red Reagent, B) on freshly-prepared hand-made cross sections from petioles (shoot) and primary roots (root) in chamomile plants. Other details are as in the Fig. 1. Additional fluorescence visualization of RNS/NO by staining with 2,3-diaminonaphthalene on freshly-prepared hand-made cross sections from primary roots (root) of chamomile plants (C): signal was transformed into pseudocolor green allowing easier comparison with Fig. 2A. Scale bar is the same as in Fig. 1.

High reactivity of ROS and NO/RNS allows assuming reciprocal changes after application of modulators. This was well evident in the present study: Cd enhanced both ROS and RNS signals (Fig. 2) while the presence of PTIO evoked depletion of NO signal (Fig. 2A) and increase in ROS (Fig. 2B) but the opposite was found after SNP co-administration. Similar responses in terms of NO changes were also observed in Cd-exposed *Medicago* roots [15] and confirm unequivocal action of the given compounds despite various species and applied concentrations. Impact of SNP and PTIO on Cd-induced changes observed here is in accordance with data from various species and indicates similar action despite different applied concentrations: SNP ameliorates negative Cd influence but (c)PTIO reverses this action in terms of oxidative stress-related parameters [15], [20], [21]. Our present data are in agreement with mentioned studies and confirm our earlier study where PTIO induced increase in ROS in Cu-exposed chamomile roots [6]. In this context of reciprocal changes between ROS and NO (depending on the modulator used), parallel elevation of ROS and NO/RNS in Cd treatment alone indicates protective action of NO under Cd stress. Surprisingly, our data are in contradiction to those observed in pea, where Cd-induced oxidative stress was related to depletion of NO and this process was evoked by changes in calcium content [5]. This may sufficiently be explained by various plant species and exposure conditions: besides, we did not observe changes in Ca level and mentioned alterations in pea plants were related to senescence induced by Cd. Senescence was not certainly visible in the present study.

We should also briefly mention staining of NO/RNS with two reagents: both 4,5-diaminofluorescein (DAF-DA, Fig. 2A) and 2,3-diaminonaphthalene (DAN, Fig. 2C) staining showed identical trend in terms of PTIO (depletion) and SNP (elevation) effect. On the other hand, DAF-DA showed extensive green signal within whole stele and on the periphery of cortex indicating potential interaction with some tissue metabolite(s). This is certainly chamomile-specific reaction because other plants we studied did not exhibit such response (Kováčik, unpublished results). It was not observed after DAN staining and signal was rather homogenous. Notwithstanding this, identical overall trends confirm usefulness of these dyes despite various chemistry of action.

#### 3.1.3. Alteration of enzymatic activities

**Figure 3:**
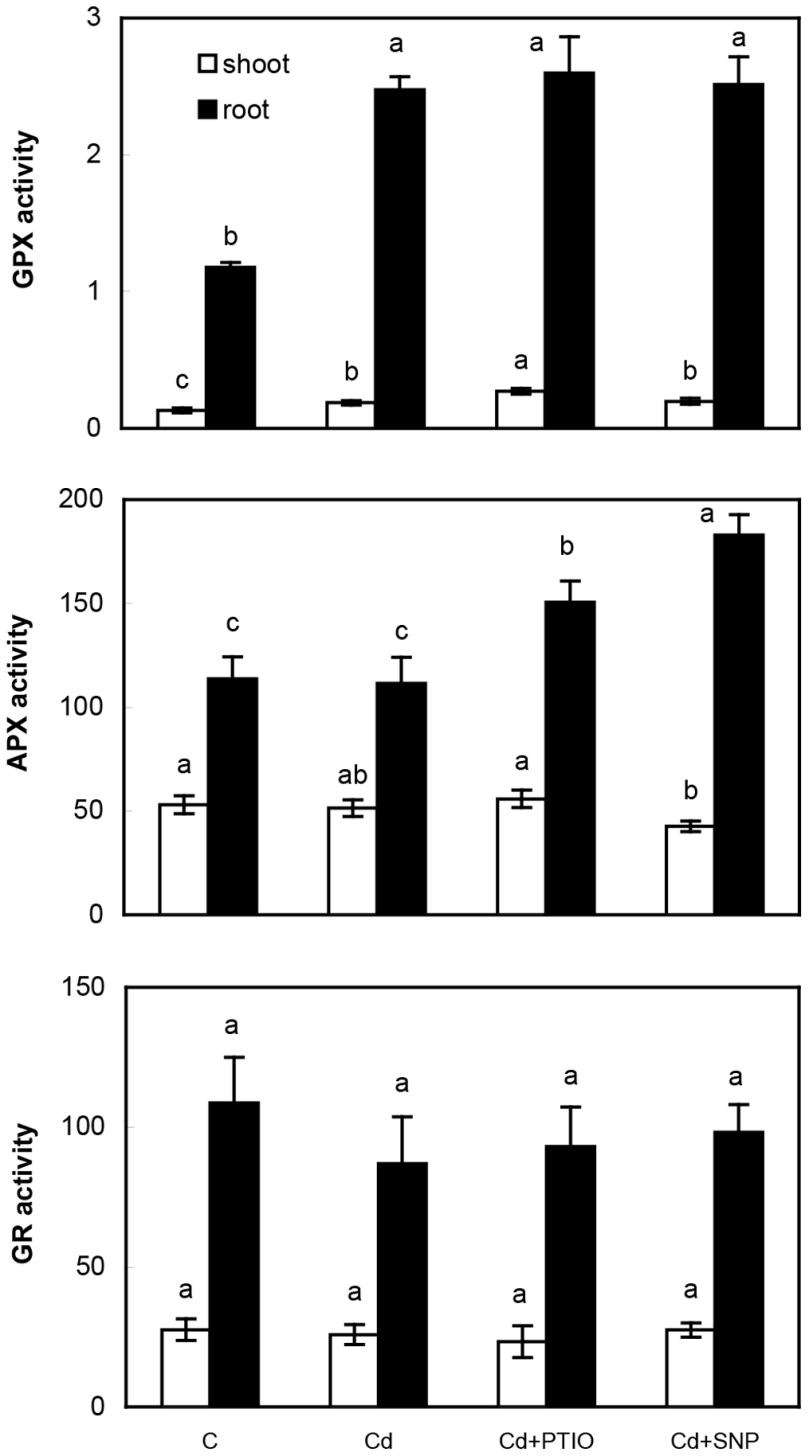
Activities of antioxidative enzymes (guaiacol peroxidase – GPX, ascorbate peroxidase – APX, glutathione reductase – GR) in chamomile plants after 48 h of exposure to various treatments as described in Fig. 1. Data are means ± SDs (*n* = 4). Values for shoot or root followed by the same letter(s) are not significantly different according to Tukey’s test (*P*<0.05). Units are µmol min^−1^ mg^−1^ protein (GPX) and nmol min^−1^ mg^−1^ protein (APX, GR). Shoot means whole above-ground biomass.

Antioxidative enzymes revealed more pronounced responses in the roots (Fig. 3) which could be inferred from their direct contact with treatment solutions. However, changes of their activities did not show reciprocal responses (while ROS and NO staining did) and rather increased after the addition of both PTIO and SNP. This is in agreement with elevated Cd accumulation and again indicates specific interaction of the given compounds with Cd. In accordance, Cu evoked various responses of PTIO and H_2_O_2_ scavenger at the level of antioxidative enzymes in chamomile roots [6] and Cd or Cu effects in terms of APX activity also differed in green alga *Scenedesmus* that was also reflected in various uptake pattern of these metals [4]. Besides, application of SNP improved growth of *Lupinus luteus* under Pb and Cd excess but antioxidative enzymes revealed discontinuous changes in relation to increasing metal concentrations [13]. This is other evidence that metal content could affect activities of antioxidative enzymes despite predicted reaction based on the applied modulator. For example, Cd elevated APX activity in *Medicago* roots and SNP evoked depletion that was related to decreased Cd content [15] while we observed the opposite (cf. Fig. 1A and Fig. 3).

#### 3.1.4. Glutathione and ascorbic acid are not directly regulated by applied NO modulators

**Figure 4:**
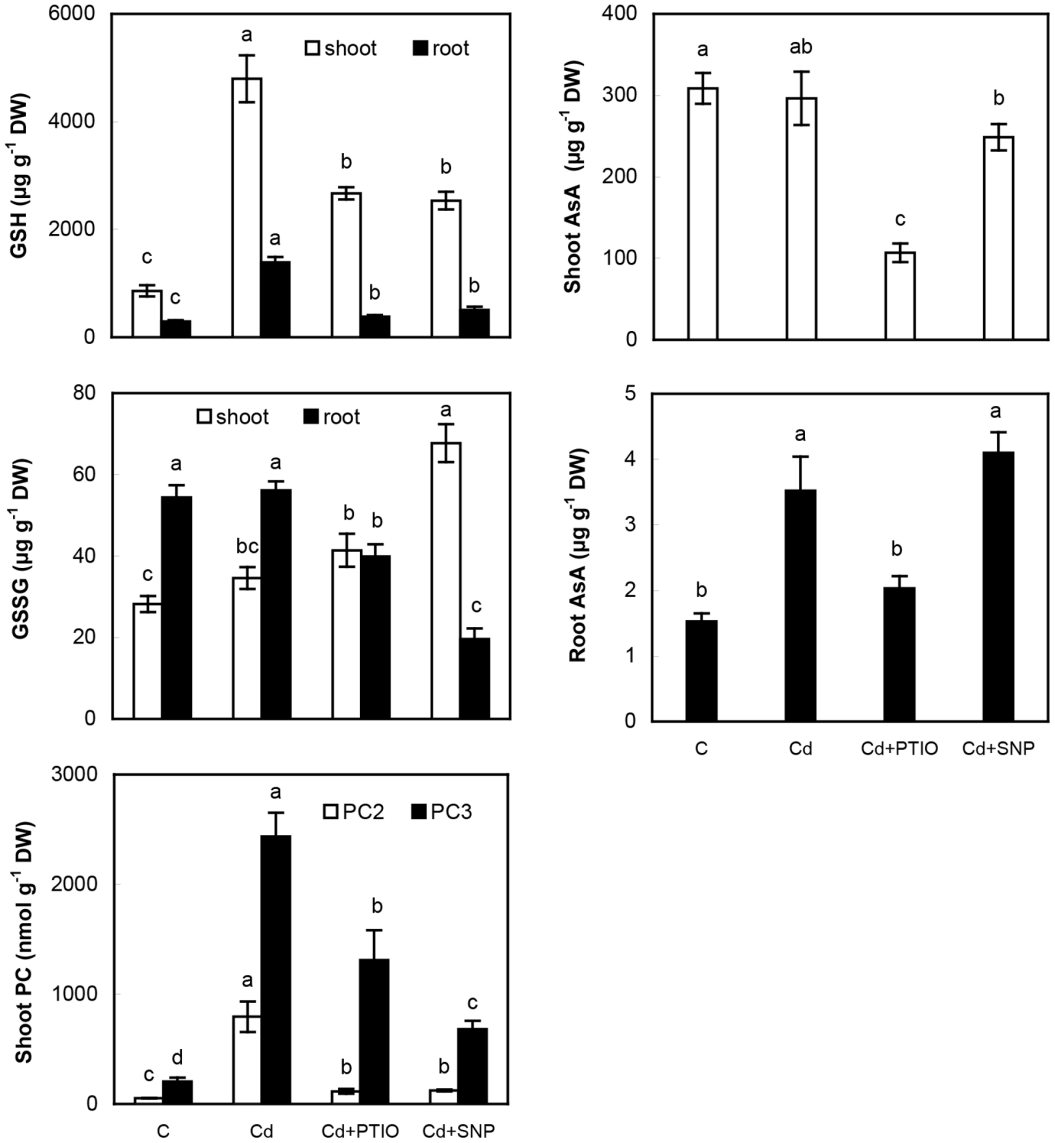
Quantitative changes of reduced and oxidized glutathione (GSH and GSSG, respectively), ascorbic acid (AsA) and phytochelatins (PC_2_, PC_3_) in chamomile plants after 48 h of exposure to various treatments as described in Fig. 1. Data are means ± SDs (*n* = 4). Values for shoot or root followed by the same letter(s) are not significantly different according to Tukey’s test (*P*<0.05). Shoot means whole above-ground biomass.

Glutathione and ascorbic acid are key non-enzymatic antioxidants [9] and GSH is also precursor for Cd-binding peptides phytochelatins [10]. We observed strong elevation of GSH and phytochelatins PC_2_ and PC_3_ in response to Cd excess while AsA content increase in the roots only (Fig. 4). Strong induction of PCs under Cd excess has previously been observed in various species [28] while GSH content was reported to decrease in barley and lettuce [30]. Surprisingly, both GSH and PCs decreased after the addition of NO modulators (Fig. 4) though various responses would be expected in terms of their impact on NO content. This is again, the most probably, related to elevated Cd accumulation. To support this assumption, PCs exhibited various quantitative changes in relation to increasing Cd concentration applied to *Linum* plants [28] leading to alteration of their free pool. Additionally, high dose of cPTIO negligibly affected GSH and AsA amounts in wheat seedlings under Cd excess [20] while we observed more expressive responses to PTIO in the present study (Fig. 4). Alteration of oxidized glutathione (GSSG) did not show identical trend in shoots and roots but considering high GSH/GSSG ratio, this certainly represents low threat to oxidative balance in chamomile. In terms of methodology, high GSH/GSSG ratio we observed is similar to that found in *Capsicum* plants [26] and it is undoubted that LC-MS/MS allows the most precise quantification currently available as discussed previously [3]. Basic values of PCs reported here are within the range observed in vascular [28] and non-vascular plants [27].

There exist also data contradicting above-mentioned results. For example, pea plants subjected to Cd exhibited induction of oxidative stress concomitantly with depletion of NO and GSH ([31] and the references therein). These data indicate complex chemistry between ROS, NO and GSH that is regulated in relation to plant species as well as stress impact. It is clear that the “power” of oxidative stress acts as the main regulatory element of this mosaic because mentioned pea plants treated with 50 µM Cd tended to senescence that was not present in our plants treated with 60 µM Cd. Inter-species comparison must therefore also consider overall growth response of the species under investigation.

#### 3.1.5. Cd- and modulator-induced changes to anatomy

**Figure 5:**
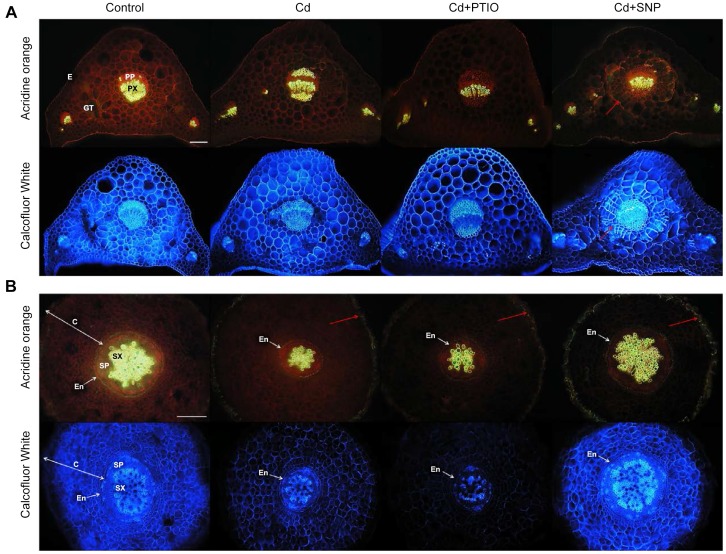
Changes in anatomy (transversal sections) of chamomile leaves (petioles, A) and roots (B) in hydroponically-cultured plants (using treatments as in the Figs. 1–4). Acridine orange stains cellulose (red fluorescence), autofluorescence of lignin, cutin, and suberin remains unchanged. Calcofluor white stains cellulose (blue). See explanation for observed changes in the discussion. E – epidermis, GT – ground tissue (parenchyma), PP – primary phloem, PX – primary xylem, C – cortex, SP – secondary phloem, SX – secondary xylem, En – endodermis. Bar indicates 200 µm. Red arrow indicates parenchyma cells with increased amount of cellulose in cell walls (A) and suberinization of the outermost cells of cortex in all treatments with Cd (B).

Anatomy of chamomile petiole is typical for this plant organ (Fig. 5A): centrally placed collateral vascular bundle consists of primary phloem (centrifugal, proto- and metaphloem cannot be distinguished) and primary xylem (vessel elements in rows, both proto- and metaxylem well distinguishable, significant portion of xylem parenchyma). Changes in response to all Cd treatments included especially alteration in the deposition of cellulose in parenchyma cells adjacent to vascular bundle (distinct in Cd+SNP variant) and changes in sclerification of elements of primary phloem (distinct in Cd-treated variant). In the control and Cd+SNP variants only individual sclerified elements of primary phloem occur and in the Cd+PTIO variant no sclerified elements of primary phloem occur. In the Cd+PTIO variant, no sclerification of parenchyma cells adjacent to protoxylem was evident. We observed not only changes in the size of parenchyma cells adjacent to vascular bundle (part of ground tissue), but also changes of deposition of cellulose in cell walls of these cells. It was suggested in leaves of Cd-tolerant willow that active storage of Cd was indicated by homogeneous cell wall thickenings with cellulose [32]. Therefore, deposition of cellulose may be connected with deposition of Cd in our study.

Root anatomy showed significant changes under Cd and NO modulators. All roots were of the same ontogenetic stage of development at the beginning of the secondary thickening with created secondary vascular tissues and preserved endodermis with Casparian strips (Fig. 5). First, changes in the rate between cortex and stele in the Cd- and Cd+PTIO-treated variants were well evident. In these cases, volume of the stele (as well as secondary vascular tissues) was significantly reduced (Fig. 5B). Suberinization of outermost cells of cortex in all Cd-treated variants was the most important noticeable anatomical change. Suberinization and lignification of the outermost cells of root cortex are described especially in monocot plants, where exodermis represents important barrier of variable resistance to the radial flow of water and solutes and usually contributes to overall resistance [33].

### 3.2. Responses of Chamomile Seedlings to Cd, PTIO and SNP Treatments

**Figure 6:**
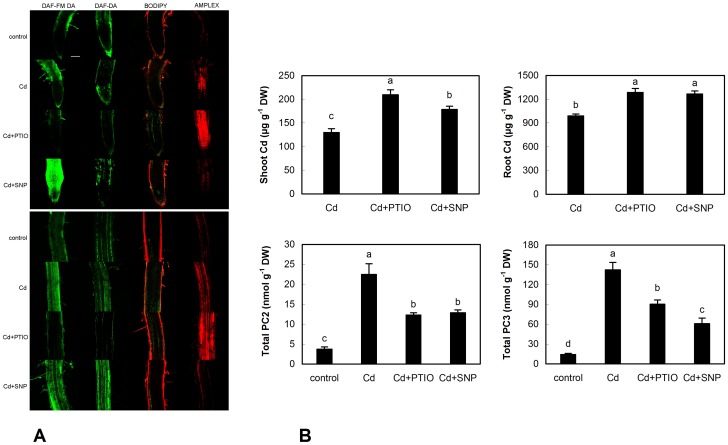
Confocal microscopy of NO changes (stained with DAF-DA and DAF-FM DA), lipid peroxidation (stained with BODIPY 581/591 C11) and general indicator of H_2_O_2_ (Amplex UltraRed) in root tips (upper panel) and upper part of roots (lower panel) of chamomile seedlings cultured on Petri dishes over 48 h with identical concentrations of Cd^2+^, PTIO and SNP as mentioned in Fig. 1. Bar indicates 100 µm (A). Changes to content of Cd and phytochelatins (PC_2_ and PC_3_) in these seedlings (B). Total PC means that whole seedlings (shoot+root) were extracted.

Impact of PTIO and SNP on Cd uptake and NO/ROS change was also verified using seedlings and treatment solutions prepared in deionised water only. Staining with DAF-FM DA showed slightly more intensive signal than DAF-DA (Fig. 6A) and in fact, DAF-FM DA is more photo-stable and less pH-sensitive than original compound DAF-DA (according to manufacturer’s web site). Differences between these reagents were not extensive (with the exception of root tip in Cd+SNP) and overall trend (decrease in PTIO and increase in SNP) was mainly observed in upper part of roots. Visualization of lipid peroxidation by Bodipy reagents revealed elevated signal in Cd and Cd+PTIO but decrease in Cd+SNP treatment that is in accordance with known protective role of NO. Amplex staining confirmed that co-application of PTIO elevates but SNP reduces ROS presence in comparison with Cd treatment alone (Fig. 6A). Though Amplex should stain hydrogen peroxide (according to manufacturer) and CellROX is not specific (Fig. 2B), trends in PTIO and SNP treatments were identical and confirm modulator-induced changes despite various plant ontogenetic stages and culture conditions. To support specificity of staining reagents, our recent study with roots of seedlings revealed that CellROX and Amplex staining did not show identical pattern under Mn excess with SNP addition [22]; this also indicates that Mn- and Cd-induced ROS are different but these staining dyes may identify differences visually. Besides, lower SNP dose (100 µM) suppressed Amplex signal more than high SNP (1000 µM) in the mentioned study with Mn [22] while 300 µM SNP in the present study suppressed Amplex signal even to control (Fig. 6A). It is therefore certain that these changes are affected by interaction of the given metal with SNP because present study confirmed increase in Cd accumulation also in both shoot and root tissue of seedlings (Fig. 6B) while SNP depleted Mn content concentration-dependently [22]. Subsequent changes of phytochelatins in seedlings (Fig. 6B) are also identical to those observed in older plants cultured in hydroponics (Fig. 4).

### 3.3. Impact of Alternative NO Modulators on Cd Uptake and Changes in Seedlings

**Figure 7:**
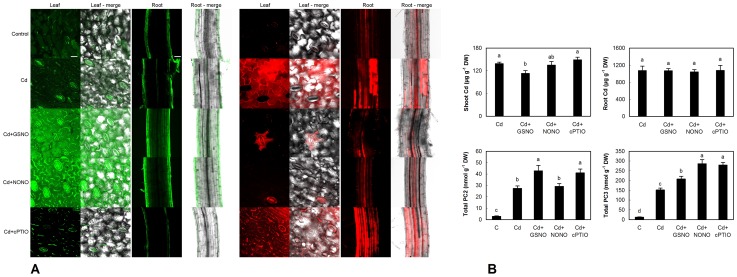
Confocal microscopy of NO changes (stained with DAF-FM DA, green emission) and general indicator of H_2_O_2_ (Amplex UltraRed, red emission) on the surface of cotyledons (leaf, first column in each panel) and in upper part of roots (third column in each panel) of chamomile seedlings cultured on Petri dishes with alternative NO modulators (S-nitrosoglutathione/GSNO - 300 µM, diethylamine NONOate/NONO - 300 µM and 2-(4-carboxyphenyl)-4,4,5,5-tetramethylimidazoline-1-oxyl-3-oxide/cPTIO - 60 µM) and 60 µM Cd^2+^ over 48 h (A). Bar indicates 25 µm for leaf and 50 µm for root. Changes to content of Cd and phytochelatins (PC_2_ and PC_3_) in these seedlings (B). Total PC means that whole seedlings (shoot+root) were extracted.

Though SNP is widely used in plant studies, its ability to generate NO in solution is substantially lower compared to other donors such as GSNO and NONOate derivatives [18]. Based on similar responses of older plants and seedlings to SNP and PTIO, we tested alternative NO donors (GSNO and DEA NONOate) and alternative NO scavenger (cPTIO) using seedlings of chamomile only. Changes to NO (elevation) and ROS (depletion) after application of GSNO and DEA NONOate were similar to those observed after SNP administration (Fig. 7A) while NO signal (using DAF-FM DA staining, green emission) was the most visible in GSNO treatment. On the contrary to PTIO (Fig. 6A), cPTIO reduced NO signal without further impact on Amplex staining that is an indication of H_2_O_2_ mainly (Fig. 7A, red emission). All these changes were well visible in both shoot (surface of cotyledons) and root tissue providing evidence about translocation of applied modulators within seedlings. We note that in comparison with fluorescence microscopy where several cell layers are visible on cross section (leading to higher brightness of photos), confocal microscopy allows to see only one layer and therefore for example stomata seem not to be present in some variants (but they are there, see merges for each staining, Fig. 7A).

Subsequent investigation revealed that alternative modulators had little impact on biochemical changes and Cd uptake: no increase in Cd content was found and even decrease in Cd+GSNO could indicate formation of Cd-GSH complexes in the treatment solution (Fig. 7B). Besides, cPTIO evoked Cd efflux in tobacco BY-2 cells even after 6 h of exposure [16] while our present data did not shown decrease in Cd amount either in PTIO or in cPTIO (Figs. 6B and 7B) suggesting involvement of exposure time and plant species in final response. Phytochelatins PC_2_ and PC_3_ also increased almost universally in all Cd co-applied compounds that fit well with no elevation of Cd content. Such responses of phytochelatins are in contradiction to SNP and PTIO application (Fig. 6B). We note no impact of applied treatments on biomass or shoot length (macroscopically visible, data not shown) probably owing to short exposure time.

Less negative impact of alternative modulators (DEA NONOate or cPTIO) in comparison with SNP and PTIO is in accordance with earlier report where SNP showed more negative impact than GSNO on antioxidative enzymes and antioxidants were also variably affected [17].

## Conclusions

Cadmium-induced changes found in this study are typically similar to those observed in other plant species under non-lethal conditions (no senescence). On the other hand, study of NO involvement in these changes using common NO modulators (SNP and PTIO) showed both expected (changes to ROS/NO signal) and unexpected responses (increase in Cd accumulation and decrease in phytochelatins or GSH). These findings (from hydroponically-cultured plants) were also fully confirmed in seedlings indicating clearly chemical (and not ontogenesis-determined) reasons. Further studies using alternative modulators (GSNO, DEA NONOate, partially cPTIO) confirmed changes to NO/ROS signal similar to those evoked by SNP or PTIO but Cd amount was not elevated and phytochelatins increased almost in all treatments. It is therefore visible that NO does not simply modulate Cd uptake and side effects occur probably owing to specific interaction of some compounds (such as SNP or GSNO) and clear physiological relationship between Cd and phytochelatins. Notwithstanding this, NO scavenger cPTIO revealed the less pronounced side impacts while similar conclusion could be done for NO donor DEA NONOate and their use in plant studies is recommended.
